# Antimicrobial Activities of Methanol, Ethanol and Supercritical CO_2_ Extracts of Philippine *Piper betle* L. on Clinical Isolates of Gram Positive and Gram Negative Bacteria with Transferable Multiple Drug Resistance

**DOI:** 10.1371/journal.pone.0146349

**Published:** 2016-01-07

**Authors:** Demetrio L. Valle, Esperanza C. Cabrera, Juliana Janet M. Puzon, Windell L. Rivera

**Affiliations:** 1 Institute of Biology, College of Science, University of the Philippines, Diliman, Quezon City, 1101, Philippines; 2 Biology Department, De La Salle University, Taft Avenue, Manila, 0922, Philippines; 3 Institute of Biology and Natural Science Research Institute, College of Science, University of the Philippines, Diliman, Quezon City, 1101, Philippines; Aligarh Muslim University, INDIA

## Abstract

*Piper betle* L. has traditionally been used in alternative medicine in different countries for various therapeutic purposes, including as an anti-infective agent. However, studies reported in the literature are mainly on its activities on drug susceptible bacterial strains. This study determined the antimicrobial activities of its ethanol, methanol, and supercritical CO_2_ extracts on clinical isolates of multiple drug resistant bacteria which have been identified by the Infectious Disease Society of America as among the currently more challenging strains in clinical management. Assay methods included the standard disc diffusion method and the broth microdilution method for the determination of the minimum inhibitory concentration (MIC) and the minimum bactericidal concentrations (MBC) of the extracts for the test microorganisms. This study revealed the bactericidal activities of all the *P*. *betle* leaf crude extracts on methicillin-resistant *Staphylococcus aureus* (MRSA), vancomycin-resistant *Enterococcus* (VRE), extended spectrum β-lactamase-producing Enterobacteriaceae, carbapenem-resistant Enterobacteriaceae, and metallo-β-lactamase-producing *Pseudomonas aeruginosa* and *Acinetobacter baumannii*, with minimum bactericidal concentrations that ranged from 19μg/ml to 1250 μg/ml. The extracts proved to be more potent against the Gram positive MRSA and VRE than for the Gram negative test bacteria. VRE isolates were more susceptible to all the extracts than the MRSA isolates. Generally, the ethanol extracts proved to be more potent than the methanol extracts and supercritical CO_2_ extracts as shown by their lower MICs for both the Gram positive and Gram negative MDRs. MTT cytotoxicity assay showed that the highest concentration (100 μg/ml) of *P*. *betle* ethanol extract tested was not toxic to normal human dermal fibroblasts (HDFn). Data from the study firmly established *P*. *betle* as an alternative source of anti-infectives against multiple drug resistant bacteria.

## Introduction

*Piper betle* L. is a tropical vine that belongs to the family Piperaceae. The plant, also known as “ikmo” in the vernacular is extensively cultivated throughout the Philippines [1], and in other Southeast Asian countries such as China, India, Sri Lanka, Malaysia, Nepal, Pakistan, Thailand and Indonesia [2]. It is popularly used in traditional medicine in these countries, which include among others, its use for treating gaseous distention, sprains, and wounds [3], as a masticatory together with lime and areca nut for oral hygiene and teeth preservation [4], as an expectorant for inflammation and infection of the respiratory tract, and for treatment of dyspnea [4, 5]. Sawangjaroen et al. showed the anti-amoebic [6] and anti-giardial [7] activities of *P*. *betle* used by AIDS patients. In addition to these bioactivities of *P*. *betle*, its antibacterial activities have likewise been extensively demonstrated in many studies [8–15]. Shitut et al. showed that the ethanol and ethyl acetate extracts of four varieties of *P*. *betle* tested had significant activities against *Vibrio cholerae* ogawa, *Staphylococcus aureus* and *Streptococcus pneumoniae*, while the hexane and benzene extracts produced moderate activities [8]. Ramji et al. demonstrated the antibacterial effects of phenolics from *P*. *betle* on obligately anaerobic oral bacteria *Fusobacterium nucleatum* ATCC 25586, *Porphyromonas gingivalis* ATCC 33277 and *Peptostreptococcus anaerobius* ATCC27337 [9]. These microorganisms produce volatile sulphur compounds responsible for halitosis. On the other hand, the crude aqueous extract of *P*. *betle* leaves caused a decrease in acid production and changes to the ultrastructure of *Streptococcus mutans*, which may have an anticariogenic effect [10]. In addition to antibacterial activities against *S*. *aureus*, the crude essential oil was also shown to have activities against the yeasts *Candida albicans* and *Malassezia pachidermatis* [11]. More recently, Subashkumar et al. demonstrated the presence of antibacterial activities of the *P*. *betle* ethanol extracts on clinical isolates of *Acinetobacter* spp., *Escherichia coli*, *Klebsiella* spp., *Proteus* spp., *Pseudomonas* spp., *V*. *cholera*, *S*. *aureus* and *Streptococcus fecalis* using the disc diffusion and well diffusion methods [12], while the same was shown by Agarwal et al. [13] on pathogenic *P*. *aeruginosa*, *S*. *aureus* and *E*. *coli*, and by Chakraborty and Shah [14] on *Streptococcus pyogenes*, *S*. *aureus*, *E*. *coli* and *Proteus vulgaris*, also using the well diffusion method. Nouri et al. [15] showed the inhibitory activities of *P*. *betle* on isolates of *S*. *aureus*, *S*. *epidermidis*, *Bacillus cereus*, *Bacillus subtilis*, *Listeria monocytogenes*, *E*. *coli*, *Salmonella typhimurium*, *Salmonella enteritidis*, *Klebsiella pneumoniae*, and *P*. *aeruginosa*. However, all these aforementioned studies did not determine the antibacterial activities of the *P*. *betle* extracts on the more novel multiple drug resistant (MDR) bacterial strains identified by the Infectious Disease Society of America (IDSA) as especially difficult to treat, and which the present study specifically addressed. In addition, extensive and careful review of scientific literature shows that studies on the effect of *P*. *betle* extracts on these MDR strains are notably lacking.

The rapid emergence and dissemination of medically-important MDR microbial strains among developed, developing and underdeveloped countries throughout the world is a realistic public health concern that need to be seriously and promptly addressed, especially if we were to consider the ease with which drug susceptible microbial strains gain resistances through mutation and acquisition of transferable resistance genes, and the positive selective pressure contributed by the indiscriminate use of antimicrobial agents in the community, health care institutions, agriculture and industry. Among the MDR strains, IDSA has identified diseases due to the following bacteria as especially difficult to treat: the Gram positive vancomycin-resistant *Enterococcus* (VRE) and methicillin-resistant *Staphylococcus aureus* (MRSA), the Gram negative extended spectrum β-lactamase (ESβL)-producing Enterobacteriaaceae, carbapenem-resistant Enterobacteriaceae (CRE), metallo-β-lactamase (MβL)-producing *Pseudomonas aeruginosa* and *Acinetobacter baumannii* [16]. All of the resistance mechanisms of these bacteria involve genetic elements that code for multiple drug resistances, and also allow insertion of additional resistance genes. At the same time, these are transferable from one bacterium to another. Enterococci have long been recognized as an important cause of endocarditis and as common causes of hospital-acquired infections [17]. They have intrinsic resistance to various antimicrobials belonging to different classes. Vancomycin was the drug of choice against these MDR enterococci, until the emergence of VRE. On the other hand, *S*. *aureus* is an opportunistic pathogen, causing substantial mortality and morbidity involving both hospital and community settings. It can cause minor to extremely lethal infections, from furuncles, scalded skin syndrome, to toxic shock syndrome, necrotizing pneumonia, endocarditis, osteomyelitis and bacteremia [18, 19]. MRSA strains are resistant to all β-lactam antibiotics, likewise making treatment a public health problem. ESβL-producing Enterobacteriaceae is similarly a global issue. These strains are resistant to all newer generation β-lactams with the oxy-imino side-chain and also to the monobactams. The carbapenem-resistant Enterobactericeae (CRE) are resistant to all third generation cephalosporins and to at least one carbapenem, the antibiotic that has been used as a final recourse to treat lethal infections caused by MDR Enterobacteriaceae species [20]. There is particular concern with regard to CRE primarily because the frequency of infections with these MDR Gram-negative bacteria is increasing, and the infections are associated with high mortality rates [21]. Both MβL-producing *P*. *aeruginosa* and *A*. *baumannii* are opportunistic pathogens that are often associated with healthcare-acquired infections with high mortality. Their ability to produce the MβL enzyme in addition to their innate resistances to various antibacterial agents makes these strains recalcitrant to treatment with commonly available antimicrobials. The widespread presence of these MDR bacteria calls for more antimicrobial studies on these medically-important bacteria using alternative sources such as plant derivatives.

Valle et al. [22] recently reported the bacteriostatic and bactericidal activities of ethanol extracts from Philippine medicinal plants on MDR clinical bacterial isolates. Results of the study showed that among the twelve (12) plants tested, *P*. *betle* L. had the greatest antimicrobial activities against both Gram-negative and Gram-positive MDR bacteria. It is in this light that the present study was conducted. It aimed to determine and compare the antimicrobial potencies of the methanol, ethanol and supercritical CO_2_ extracts of the Philippine *P*. *betle* on a larger number of MDR bacteria isolated from recent clinical cases in tertiary hospitals in the Philippines. The study did not include the isolation and identification of the antibacterial compounds. Resolute results obtained from this study would definitely strengthen the potential of *P*. *betle* as a novel and cost-effective agent against medically-important multidrug-resistant bacteria, and would be a springboard for further studies on the purification and identification of its active compounds.

## Materials and Methods

### Collection of Plant Materials

The leaves of the *Piper betle* were collected from the foot of Sierra Madre Mountain Range in the Municipality of General Nakar, Quezon province, Philippines with the following geographical coordinates: 14°47'14.5"N 121°34'06.2"E. Collection was done from September 2014 to November 2014. These were taken from *P*. *betle* trees growing abundantly in the backyards of the local residents belonging to a small community after the proper consent was secured from the owners of the lands. The identity of the plant was authenticated at the Herbarium of the Institute of Biology, University of the Philippines-Diliman, Quezon City. The leaves were washed thoroughly and then air-dried in room temperature for seven days, finely powdered and stored in a sterile airtight container for further use.

### Preparation of Ethanol and Methanol Plant Extracts

The extraction was done on the powdered dried leaves of *P*. *betle* following the methods of Basri and Fan [23] with minor modifications. The powdered plant materials in the amount of 150 g were soaked in 500 ml of absolute ethanol and absolute methanol separately for seven days with occasional stirring, and then filtered using Whatman filter paper no.1 (Whatman Ltd., England). The filtrate was concentrated under reduced pressure using a rotary evaporator at 50°C. The crude extract was collected and allowed to completely dry at room temperature. The stock solution was prepared by dissolving the dried extract in 0.2% DMSO at 100 mg/ml concentration. Hence, the 0.2%DMSO was first tested on reference strains *Escherichia coli* ATCC25922, *Staphylococcus aureus* ATCC 25923 and *Pseudomonas aeruginosa* ATCC 27853 using the disc diffusion assay to ensure that it does not have any antibacterial activities, and that whatever antibacterial activities that will be shown in the different assays can be attributed to the compounds in the *P*. *betle* itself. The ethanol and methanol extracts were also tested for the presence of contaminants before use in the different assays by inoculating 100 μL of each into three (3) thioglycollate broth and incubating the inoculated tubes at 37°C for 48 hrs and monitored for microbial growth.

### Supercritical Fluid Plant Extract Preparation

About 100 g of powered dried leaves of *P*. *betle* were loaded in the Supercritical Fluid Extractor unit using supercritical CO_2_ as extracting solvent in accordance with the methods of Nguyen *et al*. [24] with slight modifications. The CO_2_ was first liquefied before passing to a high-pressure pump. The CO_2_ liquid was heated until it reached supercritical state. The pressure and temperature were set at 15 to 20 MPa and from 35°C to 60°C, respectively. The extract was collected in a vessel while the CO_2_ passed through a rotameter at a rate of 0.5m^3^/hour before being released to the atmosphere. The process was conducted at the Iligan Institute of Technology, Mindanao State University, Lanao del Norte. As with the methanol and ethanol extracts, the supercritical CO_2_ extracts were tested for the presence contaminants before testing them for the presence of antimicrobial activities.

### Multidrug-resistant (MDR) Bacterial strains

The MDR bacterial strains used in this study, together with their resistance phenotypes are listed in Table 1. All isolates were retrieved from the Microbial BioBanks of the Makati Medical Center and Ospital ng Makati, which maintain microbial isolates collected from patients’ clinical specimens. Both are Level III training hospitals located in Makati City, Philippines. The patients from which the bacteria were isolated were anonymized, on the basis for which informed consent from the patients was not required by the respective Institutional Review Boards. All isolates were identified by automated biochemical tests using Vitek^®^MS (bioMérieux, Marcy l’Etoile, France) GP colorimetric identification card. The susceptibility patterns were determined using Vitek^®^MS AST (bioMérieux, Marcy l’Etoile, France) following the MIC interpretative standards of the Clinical Laboratory Standard Institute M100-S24 [25].

**Table 1:** Panel of MDR clinical bacterial strains used in *in vitro* antibacterial testing with *Piper betle* extracts.

MDR Bacterial Strain	Source	Resistance phenotype
MRSA 1	12/Male, Wound	SXT, FOX, OX, P
MRSA 2	69/M, Wound	FOX, OX, P
MRSA 3	42/M, Blood	SXT, FOX, OX, P
MRSA 4	35/F, Sputum	SXT, FOX, OX, P
MRSA 5	45/M, Wound	FOX, OX, P
MRSA 6	65/F, Wound	SXT, FOX, OX, P
MRSA 7	34/F, Nipple discharge	FOX, OX, P
VRE 1	45/M, Urine	AM, P, VA
VRE 2	24/F, Peritoneal fluid	AM, P, VA
VRE 3	33/F, Urine	AM, P, VA
*Escherichia coli* ESβL(+)	31/M, Urine	AM, FEP, CTX, CTZ, CRP
*Klebsiella pneumonia* ESβL(+)	25/F, Urine	AM, FEP, CTX, CTZ, CRP
*K*. *pneumoniae* CRE *(+)* 1	43/M, Blood	AM, FEP, CL, CTZ, CRO, IPM, MEM
*K*. *pneumoniae* CRE*(+)* 2	75/F, Sputum	AK, AM, FEP, CTZ, CRO, IPM, MEM
*K*. *pneumoniae* CRE*(+)* 3	52/M, Urine	AK, FEP, CTZ, CTZ, CRO, IPM, MEM
*K*. *pneumoniae* CRE*(+)* 4	59/F, Blood	AM, CL, FEP, CTZ, CRO, IPM, MEM
*Serratia marcescens* CRE(+)	75/F, Blood	AM, CL, FEP, CTZ, CRO, IPM, MEM
*Pseudomonas aeruginosa* MβL(+) 1	64,M, Blood	AK, FEP, CTZ, IPM, MEM
*P*. *aeruginosa* MβL(+) 2	75/F, Wound	FEP, CTZ, IPM, LVX, MEM
*P*. *aeruginosa* MβL(+) 3	42/F, Blood	AK, CTZ, IPM, LVX, MEM
*Acinetobacter baumannii* MβL(+) 1	53/F, Blood	AK, CTZ, IPM, LVX, MEM
*A*. *baumannii* MβL(+) 2	48/M, Urine	CTZ, IPM, LVX, MEM
*A*.*baumannii* MβL(+) 3	64/F, Blood	AK, CTZ, IPM, LVX, MEM
*A*. *baumannii* MβL(+) 4	58/F, Sputum	CTZ, IPM, LVX, MEM
*A*. *baumannii* MβL(+) 5	76/F, Blood	AK, CTZ, IPM, LVX,MEM

**MDR bacterial strains:** CRE- carbapenem-resistant Enterobacteriaceae, ESβL- extended spectrum β- lactamase, MβL- metallo β-lactamase, MRSA- methicillin resistant *Staphylococcus aureus*, VRE- vancomycin resistant enterococci

**Antimicrobial agents:** AK- amikacin, AM- ampicillin, CL- colistin, CRO- ceftriaxone, CTX- cefotaxime, CTZ- ceftazidime, FEP- cefepime, FOX- cefoxitin, IPM- imipenem, LVX- levofloxacin, MEM- meropenem, OX- oxacillin, P–penicillin, SXT- Trimethoprim-sulfamethoxazole, VA- vancomycin

### Antibacterial Susceptibility Testing

#### Disk Diffusion Method

The test bacteria were grown on sheep blood agar plates for 16–18 hr. at 35± 2°C. Well-isolated colonies were suspended in sterile 0.9% saline solution and the turbidity was adjusted against 0.5 McFarland standard to comprise approximately 1.5 x 10^8^ CFU/ml. The inoculum was swabbed on the surface of Mueller-Hinton Agar plate (Remel Inc. USA) using sterile cotton swab. Sterile 6-mm blank disks (Becton Dickinson and Company, USA) were loaded with 25 ul of diluted plant extract stock solution giving a dry weight concentration of 2.5 mg/disc. Representative antibiotic disks per bacterial strain and disks with 0.2% DMSO served as positive and negative controls, respectively. The plates were incubated at 35 ± 2°C for 16 to 24 hours. The diameters of the zones of inhibition produced by the plant extracts on test on the test isolates measured in mm.

#### Broth Microdilution Method

The minimum inhibitory concentrations (MIC) and minimum bactericidal concentrations (MBC) of the plant extracts were determined using the broth microdilution method based on Clinical Laboratory Standard Institute M07-A8 [26]. Two-fold serial dilution of each plant extract with starting concentration of 100 mg/ml was prepared using cation-adjusted Mueller-Hinton broth or MHB (Becton Dickinson and Company, USA) as diluent resulting in concentrations of 2.44 μg/ml to 10,000 μg/ml. Each set-up was carried out in triplicate in sterile 96-well microplates. Controls consisted of culture control (no plant extract), negative control (plant extract and MHB only) and reference drug controls.

The bacterial inoculum was prepared as described above for the disc-diffusion method. Ten (10) μL of the adjusted inoculum were added into each well containing 100 μL of plant extract in the dilution series, and mixed. The sealed microdilution trays were incubated at 35 ±2°C for 16 to 20 hours in an ambient air incubator.

The lowest concentration that completely inhibited the growth of the organism as compared with the growth in the culture control well was taken as the MIC of the plant extract. For a test to be valid, acceptable growth (≥ 2 mm button or definite turbidity) must occur in the latter, and no growth is observed in the negative control. On the other hand, the MBC was determined following the method described by Irobi and Daramola [27] with modifications. Wells with no visible growth in MIC assays were sub-cultured using a 10 μL loop onto a 5% sheep BAP and incubated for 16 to 20 hr at 35± 2°C. The lowest concentration of the extract that did not permit any growth was taken as the MBC.

### MTT Cytotoxicity Assay

The cytotoxicity of the *P*. *betle* ethanol extract, which proved to be the most potent among the different extracts tested in the study, was determined on normal human fibroblast cells HDFn (Invitrogen, USA). One hundered microliters (100 μL) of HDFn cells in Dulbecco’s Modified Eagle Med (or DMEM; Gibco, USA) were seeded into sterile 96-well microtiter plates. The inoculum had a density of 4 × 10^4^ viable cells/ml as determined using the Trypan Blue Exclusion method. The plates were incubated overnight at 37°C under 5% CO_2,_ and this was followed by treatment of the cells with ten (10) μL of each sample dilution. The following concentrations were tested: 100 μg/ml, 50 μg/ml, 25 μg/ml, 12.5 μg/ml, 6.25 μg/ml, 3.125 μg/ml, 1.56 μg/ml and 0.78 μg/ml. Doxorubicin in the same concentrations served as the positive control, while DMSO was used as negative control. The set-up also included wells with HDFn cells that were not exposed to the plant extract or to DMSO. These served as the untreated negative controls. The plate was incubated for 72 hours at 37°C and 5% CO_2_, after which the medium was removed and 20 μl of 3-(4,5-dimethylethylthiazol-2-yl)-2,5-diphenyltetrazolium bromide (MTT) in 5 mg/ml PBS were added into each well. After incubation at 37°C in 5% CO_2_ for 4 hours, 150 μl DMSO were added into each well. Absorbance was read at 570 nm using a microplate reader (BioTek ELx800, BioTek Instruments, USA). The resulting cytotoxicity index values (%CI) were plotted against the different concentrations of the ethanol extract and controls to obtain the corresponding linear equations for the calculation of the IC_50_ for each. Each treatment was performed in triplicate.

## Results

### Antimicrobial Activity of *Piper betle* L. leaf extracts against MDR bacteria

#### Disc diffusion Assay

In order to better evaluate the antimicrobial effectivity of *P*. *betle* leaves, three extraction methods were used to determine which of them could provide the optimum activities against MDR bacteria. The disc diffusion method was used to determine the presence of antimicrobial activities. All the 0.2% DMSO negative control discs did not produce any zones of inhibition on any of the ATCC reference isolates and MDR clinical test isolates, while the antibiotic control discs used with the MDR isolates confirmed their resistance to the test drugs as was first shown using Vitek^®^MS AST.

**Fig 1:**
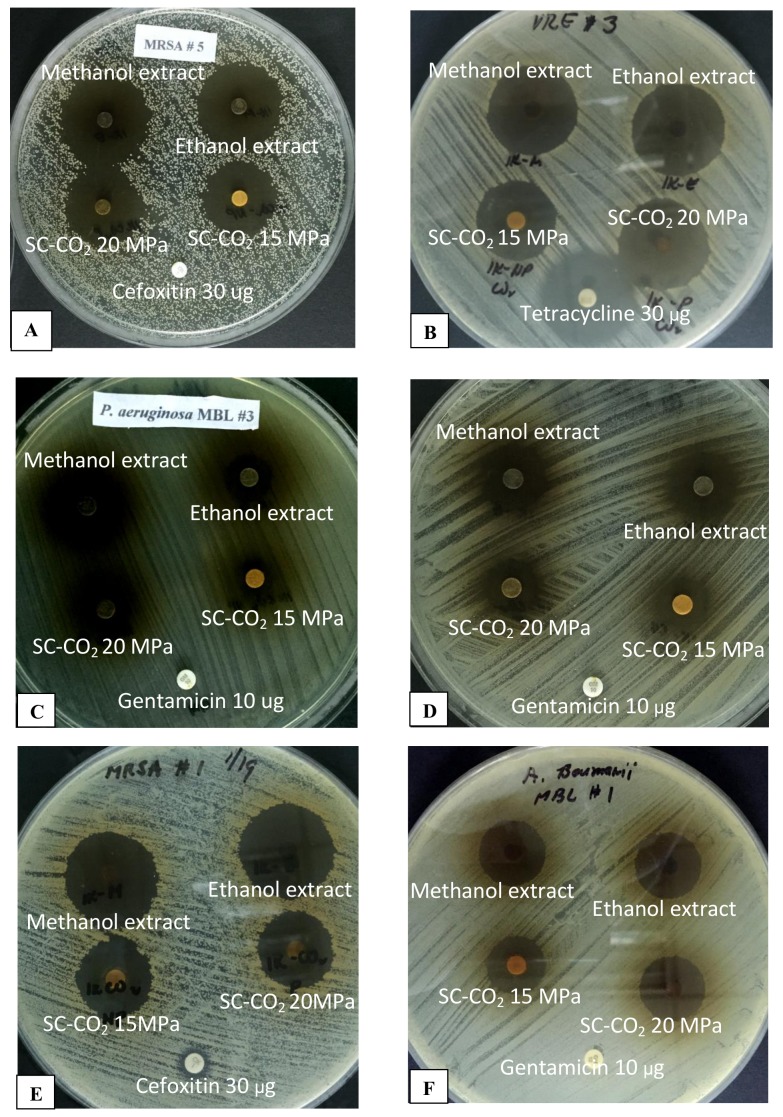
Representative plates for disc diffusion assay showing zones of inhibition produced by *Piper betle* ethanol, methanol, SC-CO_2_ 15 MPA and SC-CO_2_ 20 MPa extracts on multiple drug resistant clinical bacterial isolates, with the corresponding reference antibiotic discs. A. Methicillin resistant *Staphylococcus aureus* or MRSA 5; B. Vancomycin resistant *Enterococcus* 3; C. *Pseudomonas aeruginosa* metallo β lactamase or MβL (+) 3; D. *Klebsiella pneumoniae* carbapenem resistant Enterobacteriaceae 3; E. MRSA 1; F. *Acinetobacter baumannii* MβL (+) 1.

Table 2 shows the diameters of the zones of inhibition produced by each extraction method on the various test bacteria. All extracts produced very notable antimicrobial activities against all the bacterial strains tested. The ethanol extract demonstrated zones of inhibition ranging from 17 mm (MβL *P*. *aeruginosa* 1) to 38 mm (MRSA 5). The methanol extract showed zones of inhibition ranging from 15 mm (MβL *P*. *aeruginosa* 1) to 34 mm (MRSA 5). The supercritical CO_2_ extract at a 15 MPa pressure range exhibited zones of inhibition measuring from 11 mm to 30 mm, whereas the extract at a 20MPa pressure range gave zones of inhibition measuring from 11 mm to 33 mm. The smallest zone for each was obtained with MβL *P*. *aeruginosa* 1, and the biggest zone was with MRSA 5. Fig 1 shows representative plates of the disc diffusion assay on the different test organisms.

**Table 2:** Diameters of zones of inhibition (mm)* of *Piper betle* extracts against multidrug-resistant bacteria.

MDR bacterial strains	Ethanol Extract	Methanol Extract	SC-CO_2_ 15MPa	SC-CO_2_ 20Mpa
MRSA 1	33	32	25	25
MRSA 2	34	32	27	28
MRSA 3	28	26	21	22
MRSA 4	34	33	27	30
MRSA 5	38	34	30	33
MRSA 6	29	28	23	23
MRSA 7	30	28	23	27
VRE 1	28	26	20	24
VRE 2	25	25	15	15
VRE 3	32	32	28	31
*Escherichia coli* ESBL(+)	20	19	15	16
*Klebsiella pneumoniae* ESBL(+)	20	19	15	16
*K*. *pneumoniae* CRE(+) 1	21	21	15	16
*K*. *pneumoniae* CRE(+) 2	24	23	20	20
*K*. *pneumoniae* CRE(+) 3	23	22	16	17
*K*. *pneumoniae* CRE(+) 4	23	22	16	17
*Serratia marcescens* CRE(+)	20	19	18	18
*Pseudomonas aeruginosa* MBL(+) 1	17	15	11	11
*P*. *aeruginosa* MBL(+) 2	19	18	14	15
*P*. *aeruginosa* MBL(+) 3	28	27	12	14
*Acinetobacter baumannii* MBL(+) 1	23	22	20	22
*A*. *baumannii* MBL(+) 2	24	24	20	22
*A*. *baumannii* MBL(+) 3	24	23	19	22
*A*. *baumannii* MBL(+) 4	23	22	18	21
*A*. *baumannii* MBL(+) 5	26	25	21	24

**MDR bacterial strains:** CRE- carbapenem-resistant Enterobacteriaceae, ESβL- extended spectrum β- lactamase, MβL- metallo β-lactamase, MRSA- methicillin resistant *Staphylococcus aureus*, VRE- vancomycin resistant enterococci

SC-CO_2_ –supercritical carbon dioxide

*Zone size includes 6 mm disk

#### Minimum Inhibitory Concentration (MIC)

The MICs of the ethanol, methanol and supercritical CO_2_ extracts of *P*. *betle* for the test bacterial isolates were used to determine and compare their potencies. Table 3 shows the results of the assay. All extracts showed antimicrobial activities against both the Gram-positive and Gram-negative MDR bacteria. The extracts were more potent against the Gram-positive MRSA and VRE strains, with MICs ranging from 19 μg/ml to 625 μg/ml, and with 35% of assay results showing MICs of 156 μg/ml. On the other hand, the MICs of the extracts for the Gram negative MDR bacteria ranged from 156 μg/ml to 1250 μg/ml, with 35% and 55% of assay results showing higher MICs of 312 μg/ml and 625 μg/ml, respectively. All extracts were most potent against VRE strains 1 and 2 as shown by MICs of 19 μg/ml. Among the extracts, the ethanol, methanol and supercritical-CO_2_ 20 MPa pressure extracts had MIC values ranging from 19 μg/ml to 625 μg/ml. However, a closer look suggests that the ethanol extracts are more potent than the methanol and supercritical-CO_2_ 20 MPa pressure extracts, with the biggest percentage (36%) of MICs being 312 μg/mL, compared to MICs of 625 μg/mL for 36% of the assay results for methanol extract, and for 48% of results for supercritical-CO_2_ 20 MPa pressure extract. The supercritical-CO_2_ 15 MPa pressure extract was the least potent, with MIC values ranging from 19 μg/ml to 1250 μg/ml, the latter for 12% of the test strains.

**Table 3:** Minimum inhibitory concentrations (μg/ml) of *Piper betle* extracts for multidrug-resistant bacteria.

MDR bacterial strains	Ethanol Extract	Methanol Extract	SC-CO_2_ 15MPa	SC-CO_2_ 20Mpa
MRSA 1	156	156	625	625
MRSA 2	156	156	625	625
MRSA 3	156	156	312	312
MRSA 4	78	156	312	312
MRSA 5	78	78	312	312
MRSA 6	156	312	625	625
MRSA 7	78	156	312	156
VRE 1	19	19	19	19
VRE 2	19	19	19	19
VRE 3	156	156	156	156
*Escherichia coli* ESBL(+)	312	312	625	625
*Klebsiella pneumoniae* ESBL(+)	625	625	1250	625
*K*. *pneumoniae* CRE(+) 1	312	625	625	312
*K*. *pneumoniae* CRE(+) 2	312	312	625	312
*K*. *pneumoniae* CRE(+) 3	625	625	625	625
*K*. *pneumoniae* CRE(+) 4	312	312	625	312
*Serratia marcescens* CRE(+)	312	312	312	312
*Pseudomonas aeruginosa* MBL(+) 1	312	625	1250	625
*P*. *aeruginosa* MBL(+) 2	312	625	1250	625
*P*. *aeruginosa* MBL(+) 3	156	156	625	625
*Acinetobacter baumannii* MBL(+) 1	625	625	625	625
*A*. *baumannii* MBL(+) 2	156	625	625	312
*A*. *baumannii* MBL(+) 3	312	625	625	625
*A*. *baumannii* MBL(+) 4	312	312	625	312
*A*. *baumannii* MBL(+) 5	625	625	625	625

**MDR bacterial strains:** CRE- carbapenem-resistant Enterobacteriaceae, ESβL- extended spectrum β- lactamase, MβL- metallo β-lactamase, MRSA- methicillin resistant *Staphylococcus aureus*, VRE- vancomycin resistant enterococci

SC-CO_2_ –supercritical carbon dioxide

#### Minimum Bactericidal Concentration (MBC)

All *P*. *betle* extracts were bactericidal for all the test MDR bacterial strains. As shown in Table 4, the MBC values ranged from 19 μg/ml to 1250 μg/ml. Most were the same as the MIC values, except for the MBCs for MRSA strains 1 and 2, *E*. *coli* ESβL (+), CRE *K*. *pneumoniae* strains 2 and 3, and MβL *A*. *baumannii* strain 2, which were twofold the MIC values for some of the extracts. Although all extraction methods demonstrated antimicrobial activities, results of the disk diffusion assay, MIC values and MBC values suggest that the ethanol extract provided the highest antibacterial activity, followed by extracts of methanol and supracritical CO_2_ at 20 MPa pressure range, and the least potent were the supracritical CO_2_ at 15 MPa extracts.

**Table 4:** Minimum bactericidal concentration (μg/mL) of *Piper betle* extracts against multidrug-resistant bacteria.

MDR bacterial strains	Ethanol Extract	Methanol Extract	SC-CO_2_ 15MPa	SC-CO_2_ 20Mpa
MRSA 1	312	312	1250	625
MRSA 2	156	312	625	625
MRSA 3	156	156	312	312
MRSA 4	78	156	312	312
MRSA 5	78	78	312	312
MRSA 6	156	312	625	625
MRSA 7	78	156	312	156
VRE 1	19	19	19	19
VRE 2	19	19	19	19
VRE 3	156	156	156	156
*Escherichia coli* ESBL(+)	312	312	1250	625
*Klebsiella pneumoniae* ESBL(+)	625	625	1250	625
*K*. *pneumoniae* CRE(+) 1	312	625	625	312
*K*. *pneumoniae* CRE(+) 2	312	312	1250	625
*K*. *pneumoniae* CRE(+) 3	625	625	1250	625
*K*. *pneumoniae* CRE(+) 4	312	312	625	312
*Serratia marcescens* CRE(+)	312	312	312	312
*Pseudomonas aeruginosa* MBL(+) 1	312	625	1250	625
*P*. *aeruginosa* MBL(+) 2	312	625	1250	625
*P*. *aeruginosa* MBL(+) 3	156	156	625	625
*Acinetobacter baumannii* MBL(+) 1	625	625	625	625
*A*. *baumannii* MBL(+) 2	312	1250	1250	625
*A*. *baumannii* MBL(+) 3	312	625	625	625
*A*. *baumannii* MBL(+) 4	312	312	625	312
*A*. *baumannii* MBL(+) 5	625	625	625	625

**MDR bacterial strains:** CRE- carbapenem-resistant Enterobacteriaceae, ESβL- extended spectrum β- lactamase, MβL- metallo β-lactamase, MRSA- methicillin resistant *Staphylococcus aureus*, VRE- vancomycin resistant enterococci

SC-CO_2_ –supercritical carbon dioxide

### Cytotoxicity of *Piper betle* L. ethanol extract on normal human dermal fibroblasts (HDFn)

**Fig 2:**
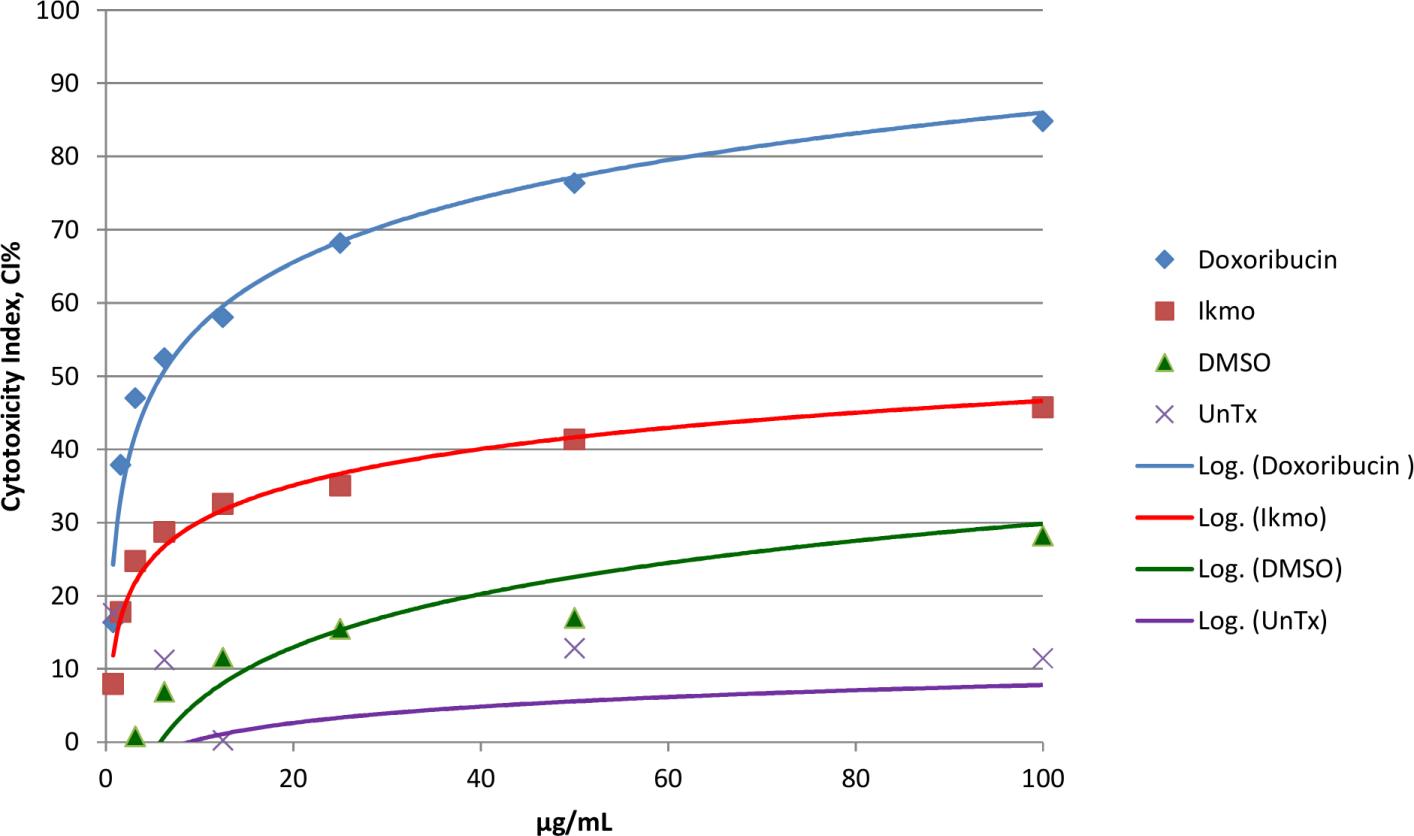
Cytotoxicity index (CI%) of *Piper betle* (or “Ikmo”) ethanol extract on normal human dermal fibroblasts (HDFn) using the MTT cytotoxicity assay. The CI% of the highest concentration tested (100μg/ml) is below the IC_50_ value, indicating nontoxicity to the cells. Positive control: Doxorubicin; Negative controls: DMSO and UnTx (untreated- no DMSO, no *P*. *betle* extract were added to the wells with HDFn).

**Fig 3:**
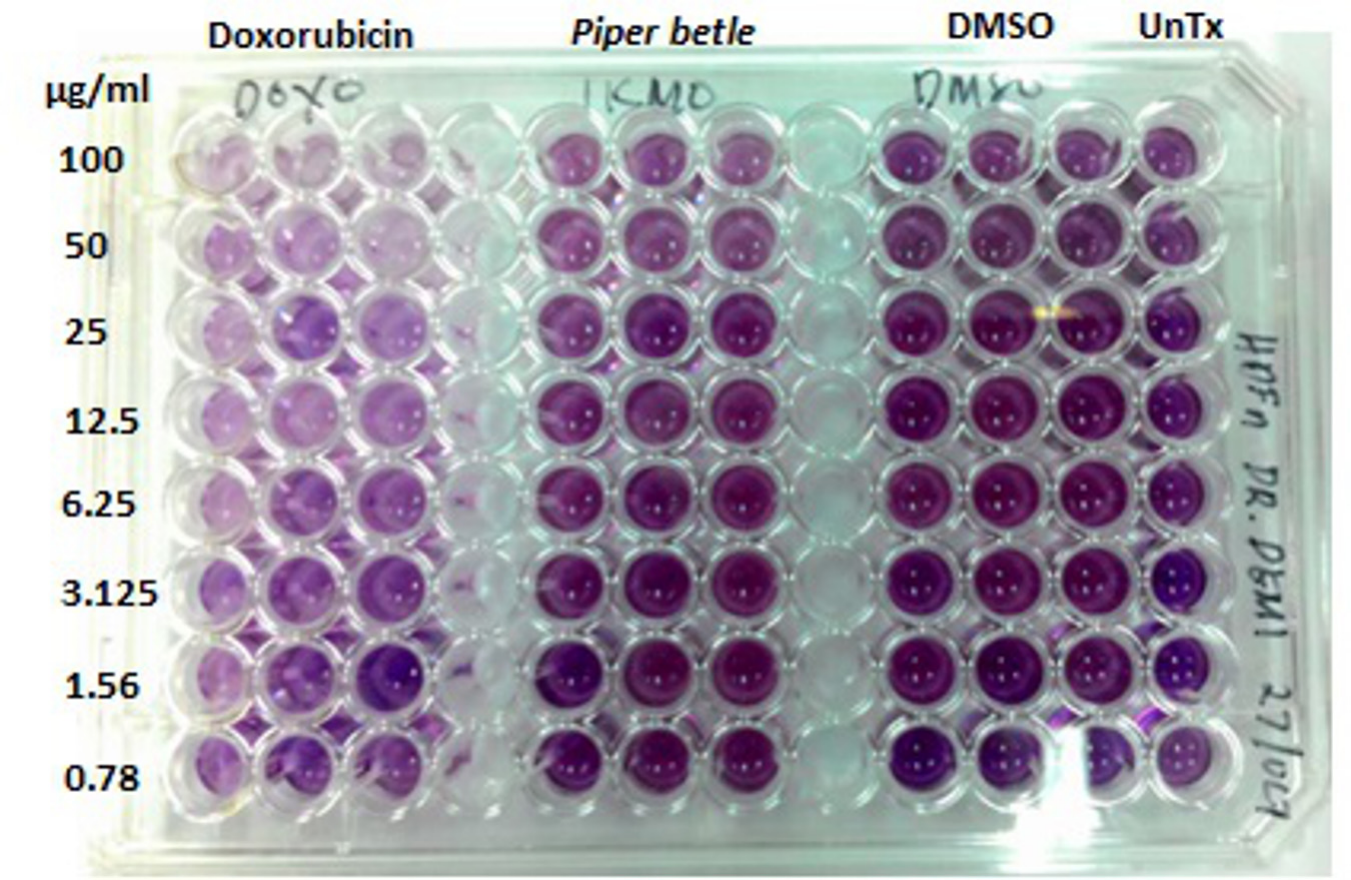
MTT cytotoxicity results for *Piper betle* ethanol extract. Positive control: Doxorubicin; Negative controls: DMSO and untreated (no DMSO, no *P*. *betle* extract were added to the wells with HDFn). Each treatment was done in triplicate.

Varying concentrations of *P*. *betle* ethanol extract were tested for their cytotoxicity to HDFn using the MTT assay. Results showed that the highest concentration of 100 μg/ml tested was not toxic to the cells as indicated its cytotoxicity index of less than IC_50_ [28] (Table 5, Figs 2 and 3). The IC_50_ for the positive control doxorubicin was 6.25 μg/ml.

**Table 5:** Cytotoxicity index (CI%) of *Piper betle* (or Ikmo) ethanol extract on normal human dermal fibroblasts (HDFn) using the MTT cytotoxicity assay.

ug/mL	Doxorubicin	*Piper betle*	DMSO	Untreated
100	84.84241	45.758183	28.22008	11.45928
50	76.36485	41.3372199	16.99763	12.84387
25	68.17878	35.0215583	15.51588	-3.77118
12.5	58.02514	32.5438756	11.55645	0.236837
6.25	52.4625	28.7058966	6.892573	11.24066
3.125	46.99702	24.7464626	0.79553	-29.714
1.56	37.8879	17.7749438	-16.9855	-19.8761
0.78	16.36607	7.98566831	-27.7464	17.58062

Positive Control: Doxorubicin

Negative control: DMSO and Untreated (no extract, no DMSO were added to the wells with HDFn)

## Discussion

With reference to the many studies conducted on the antimicrobial activities of *P*. *betle* extracts that were reviewed, this study is the first to demonstrate the antibacterial activities of the ethanol, methanol and supercritical CO_2_ extracts of the Philippine variant of *Piper betle* against various clinical isolates of bacteria with the more novel and alarming transferable multiple drug resistance mechanisms: MRSA, VRE, CRE, ESβL-producing Enterobacteriaceae and MβL-producing *P*. *aeruginosa* and *A*. *baumannii*. Results suggest that the antibacterial compounds in the crude extracts were recalcitrant to the different resistance mechanisms of these MDR isolates. This is most worthy to note since it responds to the pressing need for new antimicrobial compounds that are not targets of the drug-inactivating enzymes ESβL and carbapenamases (including the MβLs), and can still effectively function as antibacterials in the presence of the modified penicillin binding protein (PBP2A) in MRSA strains, and peptidoglycan receptors with reduced vancomycin affinity present in the VRE strains. The crude extracts did not only inhibit the growth, but were bactericidal for all the test organisms with MBCs in the range of 19 μg/ml to 1250 μg/ml. The different extracts proved to be more potent against the Gram-positive, especially for the VRE strains, than for the Gram-negative MDR bacteria. Studies have reported the role of the outer membrane in Gram-negative bacterial cell wall as a permeability barrier in conferring antibiotic resistance [29, 30, 31, 32]. Generally, the ethanol extract proved to be more potent than the methanol and supercritical CO_2_ extracts. The ethanol extract was likewise shown to be nontoxic to normal human fibroblast cells. Data of the study thus firmly show the promising potential use of *P*. *betle* compounds against both Gram-positive and Gram-negative MDR bacteria. This should spur further studies on the identification and purification of the active antibacterial compounds that would lead to eventual clinical application.
